# Signaling Pathways Regulated by Silica Nanoparticles

**DOI:** 10.3390/molecules26051398

**Published:** 2021-03-05

**Authors:** Shih-Yi Hsu, Robert Morris, Feng Cheng

**Affiliations:** Department of Pharmaceutical Science, College of Pharmacy, University of South Florida, Tampa, FL 33612, USA; shsu1@usf.edu (S.-Y.H.); rpm4@mail.usf.edu (R.M.)

**Keywords:** microarray, mechanism, nanomaterial, gene pathway, NCBI GEO, silicon dioxide

## Abstract

Silica nanoparticles are a class of molecules commonly used in drug or gene delivery systems that either facilitate the delivery of therapeutics to specific drug targets or enable the efficient delivery of constructed gene products into biological systems. Some in vivo or in vitro studies have demonstrated the toxic effects of silica nanoparticles. Despite the availability of risk management tools in response to the growing use of synthetic silica in commercial products, the molecular mechanism of toxicity induced by silica nanoparticles is not well characterized. The purpose of this study was to elucidate the effects of silica nanoparticle exposure in three types of cells including human aortic endothelial cells, mouse-derived macrophages, and A549 non-small cell lung cancer cells using toxicogenomic analysis. The results indicated that among all three cell types, the TNF and MAPK signaling pathways were the common pathways upregulated by silica nanoparticles. These findings may provide insight into the effects of silica nanoparticle exposure in the human body and the possible mechanism of toxicity.

## 1. Introduction

The prevalence of nanotechnology in medicine has greatly expanded since its emergence in the mid-twentieth century. The use of nanoparticles for gene delivery and targeted therapy allows for a reduction in effective dosage and has improved the effectiveness of many classes of drugs. The oxidized form of silicon, silica or silicon dioxide (SiO_2_), is the most abundant compound on Earth. Advantages of utilizing silica nanoparticles include their relatively low production cost, the convenience of scale-up methods, their ability to be easily modified with surface groups, and their biocompatibility [1,2,3]. Silica nanoparticles are frequently utilized for biomedical purposes including drug delivery, gene therapies, vaccine design, implants, dental fillers, and imaging studies for cancer cases [3,4]. In addition, silica nanoparticles are also used in commercial cosmetic products. Fumed silica, otherwise known as pyrogenic silica, is utilized in commercial cosmetic products in order to prevent the aggregation of cosmetic pigments [1]. Pyrogenic silica also alleviates oily skin, provides protection against damaging ultraviolet radiation, and can enhance drug penetration into the dermis layer of the skin [1]. Silica nanoparticles are also common food additives and dietary supplements in a variety of products in order to increase the shelf life as well for the enhancement of color and taste [5]. Globally, the manufacturing of silica nanoparticles has grown to be the second largest nanomaterial production in the industry [6].

However, health concerns have arisen in recent years as a result of the association between silica exposure and the toxicity to the human body. In particular, adverse health impacts induced by crystalline silica, which includes particulate silica ranging in size from 0.5 μm to approximately 10 μm, exposure have been extensively investigated. For example, crystalline silica has been linked to silicosis, a fibrotic disease characterized by severe inflammation and scarring of lung tissue, as well as other lung diseases such as lung cancer and emphysema [7]. Despite this, the underlying health complications associated with silica nanoparticles are still largely unclear and are thought to elicit different negative complications relative to crystalline silica due to the smaller size of silica nanoparticles [2].

Recent in vivo and in vitro experiments showed that silica nanoparticles may elicit cytotoxic effects in a variety of cell systems in a size and dose-dependent manner [2]. For example, in human bronchial epithelial cells, a higher number of upregulated genes in response to silica nanoparticle exposure, of which included genes involved in the inflammatory response, apoptosis, oxidative stress, and DNA damage repair, correlated with silica nanoparticles of smaller diameters [8]. Silica nanoparticles could also cause necrosis and a high degree of oxidative stress in endothelial cells [2]. This has been further corroborated in human hepatoma and neuronal cells in which a 19 nm silica nanoparticle elicited a greater cytotoxic response than a 50 nm silica nanoparticle [2]. Despite this, the underlying mechanisms in which silica nanoparticles confer toxic effects or apoptosis are not well understood.

Toxicogenomics investigates genome-wide changes (including gene/miRNA/protein expression, DNA methylation, DNA-protein binding and so on) caused by toxicants [9,10,11]. Toxicogenomics can find biomarkers of toxicity and provide insight into the mechanisms of toxicity [10]. The purpose of this study was to determine the effects of silica nanoparticle exposure on the transcriptome profiles of three different cell types as well as to elucidate the common genes and gene pathways regulated by silica nanoparticles in these cells. Three different cell types including human aortic endothelial cells, lung macrophages, and A549 lung cancer cells were analyzed in this study. The advantage of this study is to exclude the effects of backgrounds of different cells and species and find the real genes and gene pathways related to toxicity caused by silica nanoparticles. Our findings may provide insight into the mechanism of toxicity of silica nanoparticles.

## 2. Results

### 2.1. The Association between the Number of Differentially Expressed Genes Identified from Toxicogenomics Studies and Cell Survival

**Figure 1:**
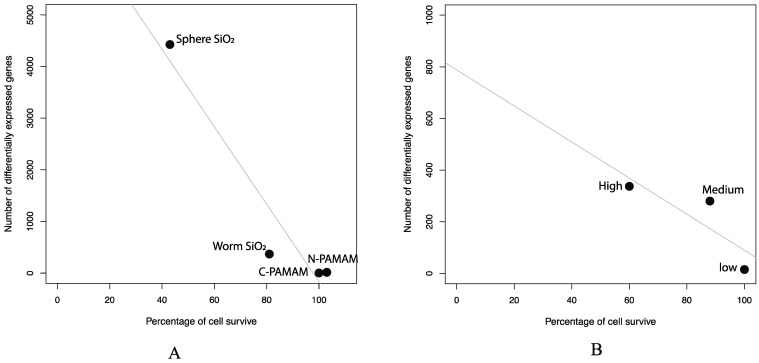
The correlation between the number of differentially expressed genes identified from toxicogenomics studies and cell survival. (**A**) In data set 1, four type of nanoparticles were compared. (**B**) In data set 2, three concentrations of 10 nm silica nanoparticles (5 µg/mL, 20 µg/mL, and 50 µg/mL) were investigated.

In toxicogenomics, the number of differentially expressed genes is a good measurement to evaluate the effects of treatment on a biological system. In our studies, we found that there is a strong negative correlation between the number of differentially expressed genes and cell survival. In the first data set, cell survival and transcriptome profiles of human aortic endothelial cells were observed after 4 h and 24 h for cells transfected with nanoparticles of various morphologies and compositions. There was a 57% (from 100% at 4 h to 43% at 24 h) decline in cell population for cells treated with spherical SiO_2_ nanoparticles at a concentration of 73 μg/mL. 4426 genes (2461 up-regulated and 1965 down-regulated genes) were found to be regulated by comparing the transcriptome of 24 h samples with 4-h samples. Transfection with SiO_2_ worm shaped nanoparticles at a concentration of 100 ug/mL resulted in a 19% (decreasing from 100% at 4 h to 81% at 24 h) cell mortality rate and 368 differentially regulated genes at 24 h, of which 250 genes were upregulated and 118 were downregulated. Transfection with either the COOH-PAMAM nano construct or NH2-PAMAM nano construct resulted in no cell mortality. A total of 15 and 2 differentially expressed genes were found for COOH-PAMAM and NH2-PAMAM material respectively by comparing the transcriptome of 4 h with 24 h. As shown in Figure 1A, there is a negative correlation between the cell survival rate and the number of differentially expressed genes, of which a greater cell mortality rate was associated with a higher number of dysregulated genes.

In the second data set, the numbers of differentially expressed genes also show a negative correlation with the cell survival percentage (Figure 1B). RAW 264.7 mouse macrophage cells were transfected with varying concentrations of 10nm silica nanoparticles. The transcriptome profiles for cells under each treatment were analyzed after 2 h. 15 genes were found to be differentially expressed upon transfection of a low concentration of 10nm nanoparticles (5 µg/mL) while 280 genes and 337 genes were found to be differentially expressed in cells treated with medium (20 µg/mL) and high (50 µg/mL) concentrations of 10nm silica nanoparticles respectively. Corresponding observed cell survival percentages for low, medium, and high concentrations were 100%, 88%, and 60% respectively. A greater number of differentially expressed genes correlated with a lower degree of cell survival. The negative correlation indicated the agreement between the toxicogenomics analysis and cell tests.

### 2.2. TNF Pathway Is the Most Enriched Pathway in the Up-Regulated Gene by Silica Nanoparticles

**Figure 2:**
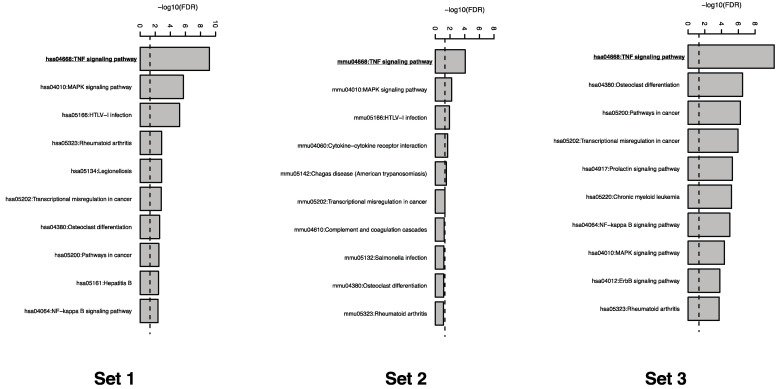
Top 10 enriched pathways identified from up-regulated genes in three microarray datasets.

For each of the three datasets, the transcriptomic profiles of silica-nanoparticle-treated cells were compared to controls. Shorter time intervals (2 or 4 h) were utilized in order to identify the pathways directly regulated by silica nanoparticle exposure. As shown in Table 1, in human endothelial cells, 2031 genes were found to be upregulated and 1662 genes were found to be downregulated in silica-treated cells relative to the control group. In the mouse macrophage cells, 178 upregulated genes and 159 genes were identified in the treatment group. Finally, 1902 genes were found to be upregulated and 1655 genes were found to be downregulated in the A549 cell line. These genes were then mapped to pathways using DAVID analysis and enriched pathways were identified. As indicated in Table 1, only a few pathways were found to be noticeably enriched for the downregulated genes in the human endothelial cells, mouse-derived macrophages, or human A549 cells. For upregulated genes, 20 pathways were found to be enriched in the silica-nanoparticle-treated human endothelial cells while 6 and 18 pathways were found to be enriched in the mouse-derived macrophages and A549 cell lines respectively. Figure 2 displays the top 10 enriched pathways generated by DAVID analysis and their FDR values.

As shown in Figure 2, for all three microarray datasets, enriched pathway analysis showed that the TNF signaling pathway had the highest fold enrichment in cells treated with silica nanoparticles. Table 2 displays the TNF signaling pathways identified in each of the three microarray datasets as well as their corresponding gene lists, *p*-values, fold enrichment values, and FDR. When comparing the transcriptome profiles of human-derived aortic endothelial cells that are treated with either silica or PAMAM nanoparticles, 29 genes involved in the TNF signaling pathway were found to be upregulated with a fold enrichment of 4.27. In RAW 264.7 mouse macrophages, nine downstream genes in the TNF signaling pathway were shown to be upregulated in cells treated with high concentration of 10 nm amorphous silica nanoparticles and had a fold enrichment of 8.70. Delivery of 9 nm silica nanoparticles into A549 non-small cell lung cancer cells resulted in the upregulation of 34 genes with a fold enrichment of 3.96.

**Figure 3:**
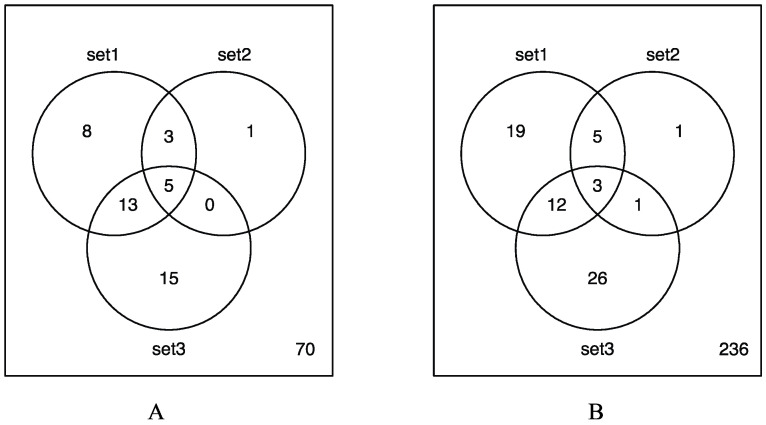
Venn diagrams of identified upregulated genes in TNF signaling pathway (**A**) and MAPK signaling pathway (**B**) that are shared between the three microarray datasets. Set1 corresponds to the human aortic endothelial cells, set2 corresponds to the lung-derived macrophages, and set3 corresponds to the A549 lung cancer cells.

**Figure 4:**
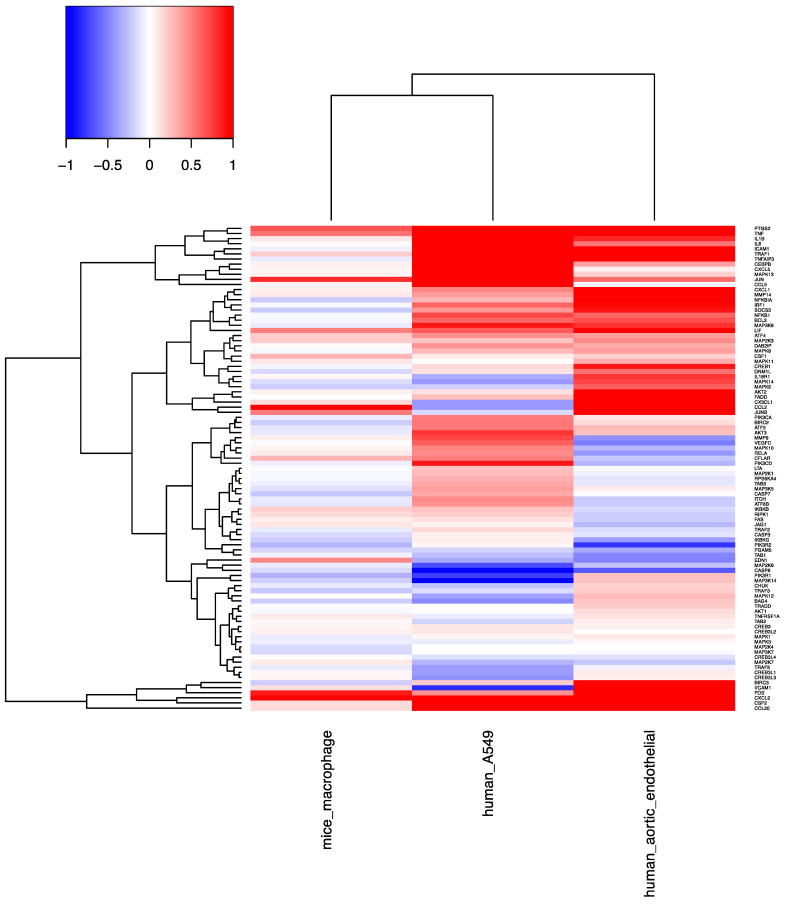
Heat map of the fold change (log2 transformed) of genes involved in TNF signaling pathways in three microarray datasets.

As indicated in the Venn diagram (Figure 3A), a significant number of genes associated with TNF-mediated signaling were found to be differentially expressed among the three microarray data sets. Out of the 115 genes implicated in the TNF signaling pathway of KEGG database, 39.1% (45 genes) were shown to be upregulated upon silica nanoparticle exposure. Among them, 18 genes were found to be upregulated in both dataset 1 and dataset 3 while eight genes were found to be upregulated in datasets 1 and 2. In addition, five genes were found to be differentially expressed in all three datasets including TNF, PTGS2, CXCL2, LIF and JUN. Figure 4 is a heatmap of gene expression change of all 115 genes in the TNF signaling pathways in response to silica nanoparticles treatment in human endothelial cells, 264.7 mouse macrophages, and A549 non-small cell lung cancer cells. Upregulated and downregulated genes are colored red and blue respectively. As indicated in the figure, the majority of differentially expressed genes of the TNF signaling pathway were subsequently upregulated by silica nanoparticle treatment as opposed to downregulated.

### 2.3. MAPK Signaling Pathway Is Also an Enriched Pathway in the Up-Regulated Gene by Silica Nanoparticles

In addition to TNF signaling, Figure 2 shows the enrichment of the MAPK signaling pathway for genes upregulated by silica nanoparticle exposures. Table 3 displays the list of differentially expressed genes identified in the MAPK signaling pathway for each of the three datasets as well as their corresponding p-values, fold enrichment, and FDR. In the first dataset, 40 genes implicated in the MAPK signaling were found to be upregulated in response to silica nanoparticle treatment, of which had a fold enrichment of 2.49. 10 downstream genes in the MAPK signaling pathway were shown to be upregulated in cells treated with silica nanoparticles with a fold enrichment of 4.20. In dataset 3, 43 MAPK signaling genes were found to be upregulated with a 2.12-fold enrichment.

As shown in the Venn diagram (Figure 3B), which portrays the number of upregulated genes in the MAPK signaling pathway that are shared between the three datasets, 67 out of the 303 genes implicated in MAPK signaling pathways (22.1%) of the KEGG database were found to be upregulated in response to silica nanoparticle exposure. Among them, 15 genes were found to be upregulated in both dataset 1 and dataset 3 while eight genes were found to be similarly upregulated in datasets 1 and 2. In datasets 2 and 3, four genes were found to be upregulated in response to silica nanoparticle treatment. In addition, three genes, specifically TNF, JUN, and DUSP16, were found to be differentially expressed (upregulated) in each of the three datasets.

## 3. Discussion

**Figure 5:**
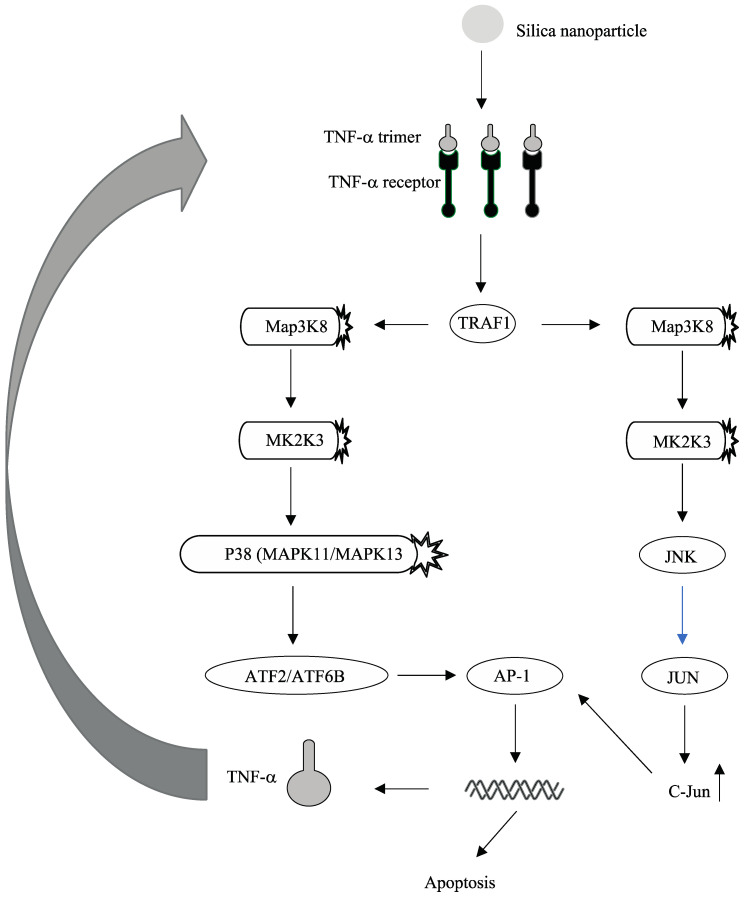
Proposed pathways regulated by silica nanoparticles conferring toxic effects.

As shown in Figure 4, several TNF-related genes (including TNF, TRAF1, Jun, and Fos) are up-regulated by silica nanoparticles in all three types of cells, which is indicative of the probable activation of the JNK-AP1 pathway by silica nanoparticles. Figure 5 represents a proposed mechanism of action that is induced by silica nanoparticle exposure and of which eventually triggers apoptosis and other cytotoxic effects. TNF-alpha is encoded by the tumor necrosis factor (TNF) gene. Trimeric soluble and membrane-bound TNF-α binds to one of two receptors in a cell-dependent manner in order to initiate a downstream signaling cascade. TRAF1 belongs to the TNF receptor associated factor (TRAF) protein family, which mediates the signal transduction from a cell’s exterior to its interior. After the binding of TNF-alpha with its receptors. TRAF1 and TRAF2 form a heterodimeric complex that subsequently activates the JNK pathway. The JNK pathway can activate the expression of pro-apoptotic genes through the recruitment of an important multimeric transcription factor: activator protein-1 (AP-1). AP-1 is a dimer in which different arrangements of c-Jun, JunD, Fos and ATF family members conjoin to elicit a variety of downstream effects [12]. Members of these protein families are all bZIP transcription factors, of which contain a leucine zipper DNA-binding motif and can form either homodimers or heterodimers. The activation of JNK and AP-1 may lead to the induction of expression of pro-apoptotic genes, which subsequently results in TNF-induced apoptosis. This observed overexpression of TNF in response to silica nanoparticle exposure has previously been validated by qRT-PCR experiments. For example, TNF-α has been shown to have a 2-fold increase (*p* < 0.01) in expression in A549 cells treated with silica nanoparticles [13]. In addition, a 2-fold increase in expression of TNF was also observed and validated by qRT-PCR in alveolar macrophages [14].

Besides the TNF-signaling pathway, MAPK pathway was also identified as an enriched signaling pathway in up-regulated genes. MAPK signaling is a cascade of phosphorylation events that leads to the activation of multiple transcription factors that regulate cell fate. In addition to TNF initiation, the MAPK signaling cascade can be triggered by ionizing radiation, certain drug classes, reactive oxygen species, growth factors, and lipopolysaccharides [15]. In most cell types, with the exception of immune cells, trimeric TNF-α binds to cell surface TNF receptor 1 (TNFR1) and initiates the activation of several downstream mitogen-activated protein (MAP) kinase-mediated signal transduction pathways.

In the case of physiological stress such as toxic substance exposure, TNF-α binding mediates apoptosis and cell survival through the use of the MAP family of kinases p38, ERKs, and JNKs. Downstream signaling mediated by MAP kinases triggers secretion of several different proinflammatory cytokines including IL-2, IL-6, and TNF-α through the transcription factor AP-1. Thus, induction of MAP kinase pathways by TNF-α binding generates a positive feedback loop that promotes apoptosis and the inflammatory response through autocrine and paracrine signaling mechanisms. As shown in Figure 5, In the case of silica-nanoparticle-induced toxicity, TRAF1 serves as the initial receiver of TNF-α signaling. TRAF1 is a MAP kinase kinase kinase kinase (MAP4K) that promotes the activation of a downstream MAP3K such as MAP3K8 through the addition of an activating phosphate group [16]. Activated MAP3K triggers activation of a downstream MAP2K such as MAP2K3, which subsequently promotes the activation of a MAPK. MAP kinases can be divided into three classes including p38, extracellular signal-regulated kinases (ERKs), and c-JUN N-terminal kinases (JNK). TNF-mediated activation of the MAPK cascade triggers the activation of p38 and JNK-regulated pathways that promote apoptosis and the inflammatory response [17]. P38 phosphorylates and activates transcription factors of the ATF family (ATF2 or ATF6B) while JNK phosphorylates downstream JUN and promotes the upregulation of c-JUN expression. Activated ATF and c-JUN then dimerize to form a functional activating protein 1 (AP-1) transcription factor that drives expression of pro-apoptotic genes and pro-inflammatory cytokines such as TNF-α and IL-6 [18]. Overexpression of pro-inflammatory cytokines such as IL-1β, CXCL2, and IL-6 have been validated by qRT-PCR in multiple studies [13,14,19]. In addition, qRT-PCR has also validated a 2-fold increase in AP-1 expression and a 4-fold increase in p38 expression in human airway epithelial cells (NCI-H292) treated with 12.5 µg/mL silica nanoparticles [20]. Downstream activation of various transcription factors by the MAPK cascade further exacerbates inflammation, promotes cell cycle arrest, and drives programmed cell death [2].

There are also several AP-1 related genes including PTGS2 (COX-2), LIF, and CXCL2, all of which were found to be upregulated in all three cell types. Allport et al. indicated that AP-1 is required for PTGS2 gene expression in amnion epithelial cell lines [21]. Concurrently, PTGS2 can also stimulate the release of mediators of inflammation including TNF-α, of which can promote further production of AP-1 through a positive feedback loop [22]. C-X-C motif chemokine ligand 2 (CXCL2) is a small secreted protein whose expression is regulated by the binding of AP-1 to the human CXCL2 promoter region. In addition, the expression of CXCL2 has been shown to be induced by TNF-mediated signaling in ovarian cancer cells [23]. In hepatocellular carcinoma, overexpression of CXCL2 has been implicated in the inhibition of cell proliferation and concurrent promotion of apoptosis through upregulation of pro-apoptotic proteins such as Bax and Caspase-7 [24]. Leukemia inhibitory factor (LIF) belongs to the IL-6 superfamily. It is a proinflammatory cytokine, of whose secretion may be stimulated by other pro-inflammatory cytokines, such as TNF-α, IL-1 and IL-6. Levy et al. has demonstrated the induction of LIF expression in mammary epithelial cells by TNF-α and AP-1 [25].

Despite the novelty of the experiment, there are some limitations that exist in the study. For example, because public datasets were utilized, we did not have direct control of the nanoparticle size or the time of treatment exposure. As a result, there were small differences in experimental design that may influence the generated results. However, the results obtained had internal consistency and reflected previously found results in the literature. Smaller nanoparticles at larger doses have consistently shown elevated cytotoxicity across multiple different metabolic and survival assays, and despite the use of different cell types, MAPK and TNF-mediated signaling pathways were found to be consistently upregulated in response to silica nanoparticle exposure. Further data acquisition and future studies are needed to further validate these findings.

## 4. Materials and Methods

### 4.1. Data Sets

As shown in Table 4, three public microarray data sets, GSE35142 [19], GSE13005 [14], and GSE53700 [13] from the Gene Expression Omnibus (GEO) database of the National Center for Biotechnology Information (NCBI) were selected to identify possible genes and pathways regulated by silica nanoparticles.

In the data set of GSE35142 [19], transcriptome analysis was performed to identify human aortic endothelial cell genes regulated by two types of nanoparticles, silica and polyamidoamine (PAMAM), at different time points (4 h and 24 h). Primary human aortic endothelial cells were acquired and cultured in Medium 200 serum prior to nanoparticle preparation. Spherical modified SiO_2_ nanoparticles were approximately 200 nm in diameter while nanoparticles with worm geometry were 200 nm in diameter and approximately 1000 nm in length. The PAMAM dendrimers differed in charge, of which G3.5-COOH nanoparticles were negatively charged and G4-NH2 nanoparticles were positively charged. Toxicity was assessed by cell count after cells were treated for either 24 or 72-h with the treated silica nanoparticles. Total RNA concentration was assessed using a Nanodrop spectrophotometer (Thermo Fisher, Wilmington, NC, USA) and 44K oligonucleotide Agilent microarrays (Huntsman Cancer Institute, Salt Lake City, UT, USA) were utilized to subsequently measure differential gene expression [19]. In our analysis, endothelial cells treated with PAMAM were chosen as the control groups based on experimental results that PAMAM nanoparticles did not cause cell death. 4 h data were chosen to evaluate the cell responses to these two nanoparticles soon after exposure to silica nanoparticles. Other studies have shown that PAMAM constructs, especially G3.5 and G4 particles at low concentrations, elicit little cytotoxic response and no change in pro-inflammatory gene expression. This notion is further supported by cell survival analysis [19].

The second data set, GSE13005 [14], was selected to investigate macrophage genes that are regulated by silica nanoparticles. In this analysis, the effects on gene expression of RAW 264.7 mouse macrophage cells by 10 nm silica at 5 (low), 20 (medium), or 50 (high) ug/mL for 2 h were investigated. Nanoparticles were obtained from W.R Grace and Company (Columbia, MD, USA) and Polysciences, Inc. (Warrington, PA, USA) The RAW 264.7 mouse macrophage cells were plated in 24-well culture plates with a concentration of 75,000 cells per well. Expression profiles were analyzed using Mouse Genome 430A 2.0 chips (Affymetrix, Santa Clara, CA, USA) in which cells were placed in 60-mm plates and then exposed to 10 nm nanoparticles for 2 h in serum-free media in order to assess immediate transcriptomic changes upon exposure [14].

The third data set, GSE53700 [13], was selected to investigate the effects on gene expression of A549 lung cancer cells by Ludox^®^ 9 nm colloidal silica nanoparticles (SM30) for 3 h. Relatively small silica nanoparticles were chosen due to the known association between smaller size and greater cytotoxicity. In addition, a short time period (2~4 h) after initial exposure to silica nanoparticles was chosen in order to determine the immediate changes in transcriptome data and resulting cytotoxic effects upon experimental treatment. A549 lung cancer cells were acquired and maintained in F12-K medium. After incubation, cells were removed from the media and incubated for 2 h in varying concentrations of silica nanoparticles. Cell survival was calculated as a ratio of the cloning efficiency of treated cells and the cloning efficiency of untreated cells. Expression profiles were analyzed using Agilent-014850 Whole Human Genome Microarray [13].

### 4.2. Microarray Data Analysis

These data sets were analyzed by the online tool GEO2R developed by NCBI [26]. The tool applies the Limma algorithm to identify differentially expressed genes [27]. The genes with adjusted *p*-value < 0.05 and absolute fold change > 1.25 were chosen. Significant differentially expressed gene lists were entered into the Database for Annotation, Visualization and Integrated Discovery (DAVID) to identify pathways that are enriched in the gene list [28]. Kyoto Encyclopedia of Genes and Genomes (KEGG) pathways were selected. The gene expression heat map was plotted using the ggplot2 package in R.

## 5. Conclusions

The purpose of this study was to determine the changes in the transcriptome profiles of three different cell types including human aortic endothelial cells, lung macrophages, and A549 lung cancer cells upon exposure to silica nanoparticles to elucidate the possible molecular mechanism of toxicity induced by these nanoparticles. An advantage of this study is that it excludes the genetic background differences among the cell types and instead, focuses on determining the common genes and subsequent pathways that are triggered by silica nanoparticle exposure. Among all three datasets, the TNF and MAPK signaling pathways were found to be the shared significantly upregulated pathways in response to silica nanoparticle exposure. Activation of the AP-1 transcription factor through p38 and JNK signaling pathways may be the primary driver of apoptosis and cytotoxic effects of silica nanoparticle exposure as well as explain the mechanism of action that confers silica-nanoparticle-mediated toxicity.

## Figures and Tables

**Table 1:** Number of up-regulated/down-regulated genes and enriched pathways (FDR < 0.05) identified from three microarray data sets.

GEO ID	Comparison	Upregulated Genes	Pathways in Upregulated Genes	Downregulated Genes	Pathways in Downregulated Genes
GSE35142	Silica nanoparticles vs. Control (PAMAM) for 4 h	2031	20	1662	1
GSE13005	Silica nanoparticles (10 nm, at high concentration) vs. Control for 2 h	178	6	159	0
GSE53700	Silica nanoparticles (9 nm) vs. Control for 3 h	1902	18	1655	3

**Table 2:** TNF signaling Pathways identified from up-regulated genes from three microarray data sets. Common genes identified from these three data sets are highlighted.

Data Set	Pathway Name	Count	*p*-Value	Genes	Fold Enrichment	FDR
1	hsa04668: TNF signaling pathway	29	5.13 × 10^−11^	TRAF1, CSF2, **TNF**, CCL2, **PTGS2**, CXCL3, **CXCL2**, NFKBIA, NFKB1, ATF2, VCAM1, **LIF**, FOS, CCL20, MAP3K8, BCL3, IL1B, ICAM1, SOCS3, MAP2K3, CREB1, MAPK11, CREB5, BIRC3, JUNB, RPS6KA5, **JUN**, TNFAIP3, SELE	4.27	6.68 × 10^−10^
2	mmu04668: TNF signaling pathway	9	7.87 × 10^−6^	**LIF**, FOS, CCL2, **TNF**, **PTGS2**, **JUN**, **CXCL2**, EDN1, JUNB	8.70	8.41 × 10^−5^
3	hsa04668: TNF signaling pathway	34	4.00 × 10^−12^	CXCL1, TRAF1, CSF2, **TNF**, **PTGS2**, MMP9, CXCL3, **CXCL2**, NFKB1, IL15, CCL5, ATF2, **LIF**, CCL20, MAP3K8, ATF6B, PIK3CA, IL1B, MLKL, PIK3R5, ITCH, AKT3, ICAM1, IL6, CEBPB, SOCS3, RELA, PIK3CD, CREB5, BIRC2, RPS6KA5, MAPK13, **JUN**, TNFAIP3	3.96	5.24 × 10^−11^

**Table 3:** MAPK signaling pathways identified from up-regulated genes from three microarray data sets. Common genes identified from these three data sets are bolded.

Data Set	Pathway Name	Count	*p*-Value	Genes	Fold Enrichment	FDR
1	hsa04010: MAPK signaling pathway	40	5.13 × 10^−11^	FGF5, **TNF**, DUSP10, NFKB1, HSPA1A, CACNB3, NFKB2, FGF12, ATF2, FOS, BDNF, RAC3, ELK4, MAP3K1, MAPT, SOS1, MAP3K8, **DUSP16**, RASGRP2, IL1B, IL1A, NFATC1, MAP2K5, LAMTOR3, MAP2K3, RELB, NR4A1, MAPK11, DDIT3, CDC25B, MAP4K3, RPS6KA5, DUSP5, RPS6KA6, DUSP2, DUSP1, RPS6KA2, **JUN**, GADD45B, MAP3K11	2.49	1.90 × 10^−6^
2	mmu04010: MAPK signaling pathway	10	5.69 × 10^−6^	HSPA8, NR4A1, DUSP2, **JUN**, JUND, GADD45B, DUSP1, FOS, **TNF**, **DUSP16**	4.20	6.07 × 10^−3^
3	hsa04010: MAPK signaling pathway	43	3.51 × 10^−6^	FGFR1, FGF5, **TNF**, PDGFB, MRAS, GNA12, DUSP10, NFKB1, NFKB2, FGF12, ATF2, KRAS, RAC2, RASGRP3, MAP3K3, JUND, MAP3K8, SOS2, **DUSP16**, IL1B, FGF2, AKT3, RASA2, LAMTOR3, TAOK1, RELA, RELB, PTPRR, STK4, DDIT3, RPS6KA5, DUSP5, MAP4K4, DUSP4, PLA2G4A, RASGRF2, MAPK13, **JUN**, MAPK8IP1, PLA2G4C, MAP3K13, DUSP8, DUSP7	2.12	4.60 × 10^−5^

**Table 4:** Three microarray data sets were used to identify the genes and gene pathways regulated by silica nanoparticles.

GEO ID	Species	Cells	Nanoparticles
GSE35142	Homo sapiens	Human aortic endothelial cells	Silica vs. control (PAMAM)
GSE13005	Mus musculus	RAW 264.7 mouse macrophage cells	Silica (10 nm) vs. control
GSE53700	Homo sapiens	A549 human lung epithelial cell lines	Silica (9nm) vs. control

## Data Availability

Three microarray data discussed in the paper are available for download from the GEO database (Access ID are GSE35142, GSE13005, and GSE53700).

## Sample Availability

Samples of the compounds are not available.
